# Using the gold mine of sleep data recorded to increase our understanding of sleep

**DOI:** 10.1093/sleep/zsae121

**Published:** 2024-05-22

**Authors:** Thomas Penzel

**Affiliations:** Sleep Medicine Center, Charité – Universitätsmedizin Berlin, Berlin, Germany

During the last 30 years we, as the sleep medicine community, had the unique opportunity to record sleep data with unbelievable precision and resolution in time and amplitude in a digital format. Recordings were performed according to the best of our knowledge using the agreed minimal quality criteria known and published in the AASM manual for the recording and scoring of sleep and associated events. The recording criteria did not change since the manuals first edition was published in 2007. Since then, we have recorded thousands of polysomnograms digitally and stored and saved the data in local repositories. Most digital systems even allow the export into a commonly agreed interchange biosignal data format. By this, the data remain to be persistent. Time resolution for the sleep EEG/EOG/EMG and ECG was set at 100 Hz and up to 500 Hz depending on equipment settings according to the polysomnography equipment. One sleep EEG recorded over one night would be 5 760 000 values (8 hours at a sampling rate of 200 Hz). Respiration signal data were recorded at 4 to 100 Hz typically. For our clinical work, only a few numbers reflecting sleep and associated events were used. The vast amount of digital data had been compressed drastically by a well-defined and largely standardized visual sleep scoring. The only time series which remained from a sleep recording was usually a graph of sleep stages. This had been created and reported in summarizing sleep recordings. The hypnogram is just a series of sleep stages across the night containing as little as 960 sleep stage codes or numbers for an 8-hour recording. And even this information had been further compressed to a few numbers like time measures (time in bed, total sleep time, wake after sleep onset, and latency to sleep), percentage measures (sleep efficiency, percentage of REM sleep, and N3 sleep), and counting measures (number of arousals, awakenings, and sleep stage transitions). We scored associated events like apnea, hypopnea events, leg and limb movements, and cardiac events. All this had been done with an enormous amount of time spent by sleep scorers checking and maintaining quality. In the end, for clinical decision-making, these simple measures were used and reported.

Realizing this, we have created a very rich arsenal of data on human sleep. Of course, these data will be only valuable if we know clinical data for the participants and patients as well. This may begin with individual anthropometric data, medication data, other diagnostic data, possibly clinical blood test values, and additional participant-reported data like complaints and reported symptoms. Often these data are available for our recorded subjects. But not very often these data are saved in a systematic database/table or electronic health record.

Clinical studies have saved these data in a systematic way according to a previously defined research question or hypothesis. The research questions may be related to new treatment options like drugs or may be related to pathophysiology questions, like understanding narcolepsy, or may be related to epidemiological questions like the prevalence of obstructive sleep apnea. Even in these clinical studies and research studies, the vast amount of sleep data recorded had been compressed to a few numbers by performing sleep scoring and summarizing events. These numbers were often similar to the numbers obtained for standard sleep reports, as mentioned above. One pathway trying to collect the raw recorded data and evaluate/exploit them was to develop automated sleep staging. An early initiative was supported by a European Union grant using pioneering digital sleep recordings following outdated Rechtschaffen and Kales montage criteria [1]. Later this early initiative turned into a startup company and then the data were no longer publicly available.

Only in recent years, computational power and software tools evolved to a capacity to treat and analyze the huge amount of data generated by sleep recordings. The easiest way to do this, are the use artificial intelligence and machine learning techniques as tools for excessive data mining. This may help us to evaluate the sleep data and get new insights into physiology and pathophysiology.

A more hypothesis-driven way could be the generation of research questions and hypotheses along the known paths of sleep microstructure. We can analyze sleep spindle density, sleep spindle frequency changes, K-complexes, the power of delta waves or alpha waves, spatial distributions over the skull, arousals, and their relations to the other physiological signals (respiration, heart rate, and pulse wave) recorded.

Now we can make use of the incredible resource of data which we have collected by using modern computational power and modern computational tools. One remarkable approach is the National Sleep Research Resource (NSRR) which has been described with its possibilities and perspectives in this issue [2]. Until now 27 systematic datasets were collected and presented publicly. We think this is just the beginning of a new age to understand sleep much better. Definitely using well-structured resources of sleep recordings can help to create a better understanding of sleep [3].

To make use of such data collection for improving knowledge and understanding of sleep and physiology, they need to follow the FAIR principles [4]. As stressed in the paper by Zhang [2], this means that the data need to be findable, accessible, interoperable, and reusable. They need to be accessible with little barriers and at no or very limited costs to encourage researchers to develop and test their hypotheses.

Of course, this excellent resource of sleep data has still some limitations. Therefore, we should promote this collection of data in order to add missing datasets, which are available in our world of clinical sleep centers. I am going to mention a number of data which could even further enrich such a collection of sleep recordings. Not many patients with insomnia disorders had been recorded with a full polysomnography. Therefore, such datasets are highly demanded. For sleep–wake rhythm disorders, actigraphy is a reduced way to record sleep. Many clinical studies used actigraphy as an accompanying measure to check whether the night in the sleep lab with polysomnography is somewhat representative of a longer period like two weeks. These actigraphy recordings were never collected systematically and are not presented in repositories. Of course, actigraphy recordings are much less standardized compared to PSG recordings. Standardization is needed for all these recordings. The same applies to pulse wave recordings/photoplethysmograms. There is no repository for pulse wave recordings and no standards for recording pulse waves. Recordings were done with different light sources, at different body locations, using different preprocessing like filtering before being stored as a signal. And then, there are many signals which are only sometimes recorded but of very high interest for phenotyping disorders and understanding pathophysiology. There are systemic blood pressure, esophageal pressure, O2 and CO2 partial pressure, core body temperature, and skin temperature.

At the same time, the data which are available at the NSRR can be used now for testing new sleep stage analysis software. They can be used to create a development, a validation, and a challenging test set. This concept relates to the original collections of recorded physiological data, the Physionet [5]. The Physionet which was built around the ECG recordings was the first such effort to present a resource of physiological data for further exploitation. Already at this initial start, sleep and sleep disorders were involved; however, reduced to the ECG and reduced to the question to recognize sleep apnea from the ECG [6, 7]. Until today there are more than 200 papers published which used this dataset to develop new algorithms to detect sleep apnea from an ECG signal. From the success of this initiative, we can learn, that high-resolution data together with new research questions can help to generate new knowledge and a new understanding of sleep physiology and sleep pathology. According to this, the NSRR is just the start of promoting sleep science. Now it is the time to think of new research questions to understand sleep and to expand the resource with good and annotated datasets which are needed for phenotyping and better understanding of sleep physiology.

## References

[1] Klösch G, Kemp B, Penzel T, et al. The SIESTA project polygraphic and clinical database. IEEE Eng Med Biol Mag. 2001;20(3):51–57. doi: 10.1109/51.932725 11446210

[2] Zhang Y, Kim M, Prerau M, et al. The National Sleep Research Resource: making data findable, accessible, interoperable, reusable and promoting sleep science. Sleep. 2024;47(7):zsae088. doi: 10.1093/sleep/zsae088 PMC11236948 38688470

[3] Mazzotti DR, Haendel MA, McMurry JA, et al. Sleep and circadian informatics data harmonization: a workshop report from the sleep research society and sleep research network. Sleep. 2022;45(6). doi: 10.1093/sleep/zsac002 PMC9189941 35030631

[4] Wilkinson MD, Dumontier M, Aalbersberg IJJ, et al. The FAIR Guiding Principles for scientific data management and stewardship. Sci Data. 2016;3:160018. doi: 10.1038/sdata.2016.18 26978244 PMC4792175

[5] Goldberger AL, Amaral A N, Glass L, et al. Physiobank, physiotoolkit, and physionet. circulation. 2000; 101: e215–e220. 10.1161/01.cir.101.23.e215 10851218

[6] Penzel T, Moody GB, Mark RG, Goldberger AL, Peter JH. The apnea-ECG database. Comput Cardiol. 2000;27:255–258.

[7] Penzel T, McNames J, de Chazal P, Raymond B, Murray A, Moody G. Systematic comparison of different algorithms for apnoea detection based on electrocardiogram recordings. Med Biol Eng Comput. 2002;40:402–407. doi: 10.1007/bf02345072 12227626

