# Correction to: Using the gold mine of sleep data recorded to increase our understanding of sleep

**DOI:** 10.1093/sleep/zsae181

**Published:** 2024-08-12

This is a correction to: Thomas Penzel, Using the gold mine of sleep data recorded to increase our understanding of sleep, *Sleep*, Volume 47, Issue 7, July 2024, zsae121, https://doi.org/10.1093/sleep/zsae121

In the originally published version of the manuscript, a reference had a title which maintained abbreviated forms. Beginning of reference 2. should read: “Zhang Y, Kim M, Prerau M, *et al*. The National Sleep Research Resource: making data findable, accessible, interoperable, reusable and promoting sleep science.[…]” instead of: “Zhang Y, Kim M, Prerau M, et al. The National Sleep Research Resource (NSRR): making data FAIR and promoting sleep science.[…]”

The emendation has been made to the article.

